# Sexual Transmission of Lyme Borreliosis? The Question That Calls for an Answer

**DOI:** 10.3390/tropicalmed6020087

**Published:** 2021-05-24

**Authors:** Natalie Rudenko, Maryna Golovchenko

**Affiliations:** Biology Centre Czech Academy of Sciences, Institute of Parasitology, Branisovska 31, 37005 Ceske Budejovice, Czech Republic; marina@paru.cas.cz

**Keywords:** Lyme borreliosis, spirochete, tick-borne disease, sexually transmitted disease

## Abstract

Transmission of the causative agents of numerous infectious diseases might be potentially conducted by various routes if this is supported by the genetics of the pathogen. Various transmission modes occur in related pathogens, reflecting a complex process that is specific for each particular host–pathogen system that relies on and is affected by pathogen and host genetics and ecology, ensuring the epidemiological spread of the pathogen. The recent dramatic rise in diagnosed cases of Lyme borreliosis might be due to several factors: the shifting of the distributional range of tick vectors caused by climate change; dispersal of infected ticks due to host animal migration; recent urbanization; an increasing overlap of humans’ habitat with wildlife reservoirs and the environment of tick vectors of *Borrelia*; improvements in disease diagnosis; or establishment of adequate surveillance. The involvement of other bloodsucking arthropod vectors and/or other routes of transmission (human-to-human) of the causative agent of Lyme borreliosis, the spirochetes from the *Borrelia burgdorferi* sensu lato complex, has been speculated to be contributing to increased disease burden. It does not matter how controversial the idea of vector-free spirochete transmission might seem in the beginning. As long as evidence of sexual transmission of *Borrelia burgdorferi* both between vertebrate hosts and between tick vectors exists, this question must be addressed. In order to confirm or refute the existence of this phenomenon, which could have important implications for Lyme borreliosis epidemiology, the need of extensive research is obvious and required.

## 1. Human Lyme Borreliosis at a Glance

Lyme borreliosis (LB) is a multisystem disorder with a diverse spectrum of clinical manifestations. It is by far the most frequent infectious arthropod-borne disease found in Eurasia and North America. Although the signs and symptoms typical of Lyme borreliosis were already described at the beginning of the twentieth century [1,2,3], the etiologic agent *Borrelia burgdorferi* was discovered much later [4,5]. One of the most frequently recorded tick-borne diseases in the Northern Hemisphere is caused by selected species of spirochaetes from the *Borrelia burgdorferi* sensu lato (s.l.) complex [6]. Currently, the complex includes 22 named species recognized internationally and countless numbers of unnamed species and strains that are not fully characterized. An accurate estimation of the importance of LB to human and animal health has not been made for multiple reasons, including significant complications in the disease diagnosis and inadequate surveillance activities [7]. Until 2013, available epidemiological data and conservative estimates kept the official numbers of diagnosed cases at about 85,000 cases/year in Europe and approximately 30,000 cases/year in the United States [8]. The year 2013 became a turning point in the history of such a controversial disease. Recognition of the impact of LB on public health came when the Centers for Disease Control and Prevention (CDC, Atlanta, GA, USA), a major world-leading epidemiological center, released the statement that about 329,000 new LB cases were diagnosed annually in the United States between 2005 and 2010, 10 times more than previously reported [9,10], and approximately 476,000 LB cases were diagnosed and treated in the USA annually during 2010 to 2018 [11]. The rise of newly diagnosed cases in Europe was recognized in a resolution of the European Parliament on Lyme disease (borreliosis) (2018/2774 (RSP)) as well, estimating almost 850,000 LB cases every year [12].

Zoonotic diseases such as LB become of concern when they spill over into the human population. LB is increasing in incidence and spreading geospatially. Recent dramatic increases in diagnosed LB cases might be due to several factors: changes in the distributional range of tick vectors; dispersal of infected ticks due to host animal migration; recent urbanization; and an increasing overlap of humans’ habitat with that of wildlife reservoirs and ticks vectors of *Borrelia*. The involvement of other bloodsucking arthropod vectors and/or other routes of transmission (human-to-human) of the causative agent has been speculated to be contributing to increased disease burden. Lyme borreliosis is also one of the most controversial diseases in the history of medicine [13]. The causative agent of LB, spirochetes from the *Borrelia burgdorferi* s.l. complex, is transmitted from infected tick to the vertebrate host, including humans, during tick feeding. This mode of transmission was established when the connection between LB, ticks and spirochetes was first discovered [4,14,15] and is accepted as the primary mode of bacterial transmission. Yet, this does not exclude other minor modes of infection, e.g., sexual, congenital or with blood transfusion. If tick feeding is the only gateway for *Borrelia* to enter the host, then the number of tick bites must be enormous. For example, the number of confirmed tick bites in the Netherlands was estimated to be 495 per 100,000 population [16], which translates into approximately 1,500,000 tick bites in the USA per year. Based on the calculation that only 2% of tick bites result in infection [17], close to 15,000,000 tick bites per year would be needed to achieve the recently released CDC LB infection prevalence. Applying the same calculation to the present population of Europe, where the great heterogeneity in LB distribution is well known and the number of recorded cases on average is recognized as 100 per 100,000 population (with the incidence rate per country from 0.01 to 350 cases per 100,000) [18], the amount of tick bites might be close to 37,000,000 per year to reflect the estimations of the European Parliament. Such a high tick bite number suggests that other modes of LB transmission may occur. One such mode could be sexual transmission. For some reason, the question about the possibility of the existence of human-to-human *Borrelia* transmission is underdiscussed and it is practically impossible to find a study that deals with the sexual transmission of *Borrelia* among humans, or the studies that support the conclusion that this route of transmission does not exist.

## 2. Sexual Transmission of Spirochetes among Hosts

Lyme borreliosis spirochetes are a highly specialized bacteria basically circulating between vertebrate hosts and invertebrate vectors. Spirochetes are transmitted to vertebrates, including humans, via a tick bite together with tick saliva which contains a cocktail of immunomodulatory molecules. *Borrelia burgdorferi* is a complex organism with similarity to *Treponema pallidum* subspecies *pallidum*, the causative agent of venereal syphilis, where the sexual transfer of the pathogen is abundantly documented [19,20]. Syphilis is a classical sexually transmitted disease distributed worldwide. Lyme borreliosis is a multisystem infectious disease caused by spirochetes from the *Borrelia burgdorferi* sensu lato complex transmitted by ticks, and is the most frequent zoonosis worldwide. Both *Treponema* and *Borrelia* belong to the order *Spirochetales*, which is pathogenic to humans, and both pathogens are bound by ancient ancestry, similar morphology, the protean nature of the long-term disease that they cause and a chronically infected state in the untreated hosts, including humans [21]. Syphilis and LB have similar etiologic, clinical and epidemiologic characteristics. Both are globally distributed multisystem infectious disorders. Their clinical course can be divided into stages and antibiotic therapy is similar as well. The taxonomical relationship between *Treponema* and *Borrelia* could also explain the congenital manifestations well known in syphilis, and suggested in LB [22]. Syphilis and Lyme borreliosis are both chronic, multistage infections that are characterized by periods of remission and exacerbation. Underlying their protean nature is the remarkable ability of both of these spirochetes to persist for prolonged periods, despite the humoral and cellular responses that they elicit in infected individuals [21,23]. LB spirochete has the ability to form pleomorphic forms, such as cysts or round bodies, or to participate in protective biofilms [24,25,26,27,28]; it is able to disseminate into multiple privileged sites such as joint, eye, synovium, heart or brain. The genital tract could as well harbor the infection and lead to vector-free transmission of spirochete between hosts, including humans.

The possibility of transmission of the LB spirochete without the tick vector was discussed as early as 1986 [29]. Burgess and colleagues published their findings of direct contact transmission of *B. burgdorferi* between infected and uninfected wild mice, *Peromyscus leucopus* and *P. maniculatus*. Uninfected mice in contact with infected mice, of both species, developed antibodies to *B. burgdorferi* by day 14 after exposure to infected cage-mates. Further, spirochetes were recovered from the blood of one contact-exposed *P. maniculatus* 42 days after initial contact [29]. Subsequent studies by Wright and Nielsen demonstrated the susceptibility of mice to oral infection with *B. burgdorferi* and the transmission of spirochetes from infected males to uninfected females by direct contact [30]. On the other hand, studies on Lewis rats and Syrian hamsters failed to confirm the existence of either the sexual transmission of LB spirochetes or transmission by non-sexual direct contact in these two animal models [31,32].

Canine models added further evidence for the possibility of vector-free transmission of *Borrelia*; an uninfected female dog seroconverted from negative to positive after sexual intercourse with an experimentally infected male dog, indicating the possibility of transmission of spirochetes in semen. Furthermore, *Borrelia* DNA was detected in tissues of fetuses from the following pregnancy [33].

The question of possible sexual transmission between sexually active partners was discussed by Bach in 2001 [34]. Working with genital fluid samples (semen of male LB patients and vaginal swabs of LB female patients) Bach detected LB spirochetes by microscopy and culture in 40% of uninfected sexual partners or those with no history of previous tick exposure. According to his observations, sexually active couples seemed to have a propensity for antibiotic failure, interpreted as reinfection through sexual contact. Almost a decade later, another group conducted *Borrelia* cultures on human semen and vaginal secretions [35]. Spirochete DNA was amplified by PCR from cultures of genital secretions from 11 out of 13 patients diagnosed with Lyme borreliosis, and motile spirochetes were observed in genital culture concentrates from 12 of 13 LB patients by light- and dark-field microscopy. All cultures were negative for treponemal spirochetes. Molecular hybridization and PCR testing confirmed that spirochetes isolated from genital secretions of sexually active couples having unprotected sex were strains of *Borrelia* [13,35].

Another interesting study published in 2003 documented the largest group of chronically ill *Borrelia* seropositive patients in a “zoonotically” non-endemic LB area [17]. Harvey and Salvato challenged the CDC definition of LB as an exclusively zoonotic disease, proposing a significantly altered model of human *B. burgdorferi* infection that included “Lyme disease”—a zoonotic disease primarily located in limited geographic areas—and “Epidemic Borreliosis”—a disease spread directly between humans with a global geographic distribution, greater prevalence and more variable clinical presentation [17]. Using these assumptions, the authors modeled the prevalence of human *B. burgdorferi* s.l. infection under three transmission models: (1) if zoonotic transfer is the only way that exists then calculations predicts that 2% of humans are infected; (2) if congenital transfer is combined with vector transfer over at least a millennium, then the number of infected humans will reach 6.5%; and (3) if vector, congenital and sexual transfers are combined for at least one millennium, then we can expect cca. 15.5% of a population to be infected with LB spirochete. Even though primary transmission is presumably via the tick vector, any human-to-human infection will increase infections in the human population over time [17]. The use of animal models would be a significant step ahead in determining if viable LB spirochetes survive in the genital tract and can be really transmitted between sexually active partners. The specific questions that need to be addressed are: (a) can only selected pathogenic LB species be transmitted—is transmission species-specific?; (b) is transmission uni- or bi-directional with regard to sex—is transmission from males to females only?; (c) could transmission occur only under specific conditions, such as the involvement of specific LB species or the presence of multiple infection in a sexual partner? Whatever the conclusions, this topic deserves deep attention as the results might elevate the complexity of dealing with LB if is it both a vector-borne disease and a sexually transmitted illness.

Mentioned studies conducted on animal models and those involving humans indirectly suggest that LB spirochete may be sexually transmitted, as in the case of the causative agent of venereal syphilis [13,21]. The possibility to conduct studies on direct transmission of LB spirochetes in humans, as in the “volunteer” experiment performed on syphilis patients [36,37], might not even be considered now due to its unethical nature. This means that animal models are even more important and the original studies involving animal models should be reproduced. Using animal models it could be possible to confirm if the viable spirochetes observed in genital secretions are not just able to disseminate into and to survive in the genital tract, but to be really transmitted between sexually active partners during sexual intercourse.

## 3. Sexual Transmission of Spirochetes among Tick Vectors

While sexual transmission of LB spirochetes in vertebrate hosts, including humans, is a rather speculative and controversial issue, sexual transmission of bacterial pathogens is a well-known phenomenon in arthropod vectors, first described in soft ticks (*Ornithodorinae*) for relapsing fever *Borrelia* [38,39,40]. Male *Ornithodoros (Pavlovskyella) erraticus* infected with *Borrelia crocidurae* transferred the spirochetes to females during copulation. After the first and second gonotrophic cycles, spirochetes were observed in 23 and 37% of the females, respectively.

Later the sexual transmission of *B. burgdorferi* s.l. spirochetes was observed in *Ixodes persulcatus* ticks, confirming the fact that female ticks may acquire selected spirochete species directly from their infected male partners. The infection rate among ticks maintained as sexual pairs was 1.75–2.00 times higher than among the ticks maintained singles, indicating that borrelia exchange between sexual partners was the result of a venereal or omovampiric (cannibalistic) mode of spirochete transmission [41,42].

The same authors revealed the presence of *B. afzelii, B. garinii* or a mixture of both spirochete species in field-collected *I. persulcatus* ticks [42]. One hundred thirty eight ticks were kept as pairs (69 pairs) that copulated during the 3–4 weeks maintenance period. An interesting observation was published, showing the existence of sexual transmission of *B. garinii* from infected male to uninfected female ticks, while no sexual transmission from infected females to uninfected male ticks was detected in the case of the presence of single spirochete species infection. The sexual transmission from male to female was confirmed in the case of the presence of double-infection with *B. garinii* and *B. afzelii* in the male tick, confirming the male’s ability to infect the partner female with a single spirochete species, as well as with present double species. None of the tick females which carried *B. garinii* and *B. afzelii* at once were able to transmit either a single or a double species to the tick male partner [42].

Contrary to *B. garinii*, another major spirochete species, *B. afzelii*, was not transmitted between tick partners who carried this species as a single infection, neither by males nor by females. However, when *B. afzelii* was present in the tick in co-infection with *B. garinii*, transmission between the partners occurred, and the male tick with dual infection was able to transmit *B. afzelii* to uninfected female. This may lead to the conclusion that either *B. afzelii* is not transmitted efficiently by *I. persulcatus* ticks, or that sexual transmission of *B. afzelii* is supported by another spirochete species present in the same vector; in the discussed case, by *B. garinii* [43].

## 4. Issues to Consider

The rapidly increasing number of recognized LB cases worldwide suggests the possible existence of other modes of transmission of LB spirochete, in addition to the traditionally recognized vectoring by ticks. Lyme borreliosis shares many features with the other recognized human spirochetal diseases for which the existence of venereal transmission is abundantly documented (for example, venereal syphilis). These similarities include: (1) skin or mucous membrane as an entry point; (2) spirochetemia early in the course of disease, with wide dissemination through tissue and body fluid; (3) multiple stages of disease, often with intervening latent periods; (4) survival of viable spirochetes in human genital secretions; and (5) tropism for skin, neurologic or cardiovascular tissues.

Different species of LB spirochetes, as well as different strains of selected species, exhibit considerable genetic heterogeneity, locally and globally. They also possesses different invasive potential. The *ospC* gene that encodes the highly polymorphic outer surface protein C defines strain invasiveness in vertebrate hosts, including humans. Analysis of a diverse group of *B. burgdorferi* s.s. strains based on this gene revealed a significant separation of types that have the potential to develop invasive disease and are involved in disseminated LB around the world, and those that are maintained in enzootic cycles [44]. If highly invasive strains naturally coexist with the non-invasive, serious consequences of the disease might occur due to the virulent variants of spirochetes, their evolution and polymorphism. Do the genetic variations in the spirochete species control the transmission route or are they involved in its constant evolution? It is possible that sexual transmission of spirochetes could be restricted or more prevalent in some strains or spirochete species.

Out of 22 recognized species from the *B. burgdorferi* s.l. complex, ten were confirmed to have a pathogenic potential in humans: *B. afzelii, B. bavariensis, B. bissettii, B. burgdorferi* sensu stricto, *B. garinii, B. kurtenbachii, B. lusitaniae, B. mayonii, B. spielmanii* and *B. valaisiana*. Nevertheless, the vast contribution to human LB worldwide still belongs to *B. afzelii*, *B. burgdorferi* sensu stricto and *B. garinii*. Different *Borrelia* species possess different organ tropisms and preferentially cause distinct clinical manifestations of disease. Lyme arthritis is the most common musculoskeletal symptom resulting from *B. burgdorferi* s.s. infection. About 60% of untreated patients with erythema migrans (EM) experience brief or sustained attacks of arthritis in America [15]. In contrast, only 3 to 15% of LB patients suffer from arthritis in Europe [45], where *B. garinii* and *B. afzelii* are more frequently recovered than *B. burgdorferi* s.s. Serotyping studies of isolates from Europe reveal a remarkable correlation between neuroborreliosis and infection with *B. garinii*. Nevertheless, *B. burgdorferi* s.s. and *B. afzelii* can also be associated with neurological manifestation; however, not at such a high incidence [46,47]. *B. afzelii* in humans seems to have a tropism for skin, since it preferentially causes EM, lymphadenosis benigna cutis [48] and acrodermatitis chronica atrophicans (ACA) [49]. *B. afzelii* is the predominant, but not the exclusive, etiologic agent of ACA; *B. garinii* has also been detected in patients with ACA [47,50]. Although ACA has been rarely reported in the United States, it can be observed in approximately 10% of European cases of LB [51]. A connection of *B. bissettii* with cardiovascular manifestations of LB was revealed in European patients [52,53]. The evidence of individual genetic pathways that lead to different tissue tropisms in closely related species of Lyme borreliosis spirochetes is the best proof for a genetic basis that defines transmission route. Due to existing tissue tropism or preferential sites of dissemination, it is reasonable to expect contrasting transmission modes of selected LB spirochete species.

Another issue that indirectly supports the idea of possible unequal sexual transmission of different species from the *B. burgdorferi* s.l. complex is the connection between the geographic predominance of specific species and the annual incidence of LB cases in different countries. While Slovenia or Austria, where *B. afzelii, B. garinii, B. bavariensis, B. bissettii* and *B. burgdorferi* s.s are abundant, belong to highly endemic LB regions with an average of 206 and 135 annual cases per 100,000 population, respectively, their neighbors, Italy, Portugal or the European part of Turkey, where *B. lusitaniae* and *B. garinii* represent the major circulating spirochete species, report annually approximately 0.02, 0.04 and 0.01 cases per 100,000 population, respectively [8,54,55]. Is it possible that such a drastic difference is determined by the ability of selected spirochete species to succeed in both traditionally recognized vector-secured and controversial vector-free transmission?

An open discussion and experimental confirmation of the existence of vector-free spirochete transmission could have a significant impact in challenging the Lyme borreliosis paradigm. Confirmation or refutation of the possibility of sexual transmission of LB spirochetes must be supported by sophisticated immunological and molecular methods. For example, analysis of pathogen genetics and their persistence in the hosts; the role of host-shifts in the emergence of human LB; comparison of bacterial strains isolated from sexually active couples with the purpose to confirm spirochete clonality; and metagenomic analysis of semen or vaginal secretions to provide further support for the hypothesis of the possible existence of sexual and non-sexual transmission of spirochetes. Whatever the results will be, such research is necessary to promote human health and limit the increase in Lyme borreliosis cases around the world. “In the context of human diseases, there is a remarkable lack of understanding “why” and “when” different transmission modes are likely to evolve, and whether changed circumstances following pathogen entry into a human population would result in the evolutionary amplification of a transmission pathway” [56]. Is Lyme borreliosis another sexually transmitted infection (STI)? That is the question that calls for an answer.
